# Increased oxygen demand during exercise as a stimulus for neuroprotection: A working hypothesis

**DOI:** 10.4103/NRR.NRR-D-25-00864

**Published:** 2025-12-30

**Authors:** Johannes Burtscher, Robert Motl, Erich Hohenauer, Luis Santos, Atbin Djamshidian, Hannelore Ehrenreich, Florian Krismer, Klaus Berek, Martin Burtscher, Katharina Hüfner, Martin Kopp

**Affiliations:** 1 Department of Sport Science, University of Innsbruck, Innsbruck, Austria; 2 Department of Psychiatry, Psychotherapy, Psychosomatics and Medical Psychology, University Hospital for Psychiatry II, Medical University of Innsbruck, Innsbruck, Austria; 3 Department of Kinesiology and Nutrition, University of Illinois Chicago, Chicago, IL, USA; 4 Rehabilitation and Exercise Science Laboratory, Department of Business Economics, Health and Social Care, University of Applied Sciences and Arts of Southern Switzerland, Landquart, Switzerland; 5 Department of Neurosciences and Movement Science, University of Fribourg, Fribourg, Switzerland; 6 Departmento de Ciencias de la Educación, Universidad de Oviedo, Oviedo, Spain; 7 Department of Neurology, Medical University of Innsbruck, Innsbruck, Austria; 8 Experimental Medicine, Central Institute of Mental Health, Medical Faculty Mannheim, Heidelberg University, Mannheim, Germany

**Keywords:** cardiorespiratory fitness, exercise, hypoxia inducible factor, mental health, mitochondria, neurodegeneration, neurological disorders, neuroprotection, oxygen, physical activity

## Abstract

Aerobic (endurance) exercise training protects from age-related neurological and psychiatric diseases. The bi-directional signaling between tissues directly involved in aerobic exercise, such as skeletal muscle and the brain, is well established; however, the precise mechanisms by which exercise benefits the brain remain elusive. We summarize the role of hypoxia (reduced oxygen availability) signaling as a potential mediator of exercise outcomes on the brain. The increased oxygen demand in organs such as skeletal muscle and heart during aerobic exercise induces hypoxia responses, including the activation of hypoxia-inducible factor pathways. These responses promote adaptations leading to improved oxygen transport, mitochondrial functions, and oxidative stress management in the brain and thereby counteract central pathological developments associated with neuropsychiatric and neurodegenerative diseases. Passive hypoxia exposures can similarly improve brain functions; we provide an extensive overview of the existent literature on that topic. We conclude that the combination of aerobic exercise and ambient hypoxia can result in synergistic and/or additive positive outcomes in the brain. However, the dose of either stimulus and individual resilience/vulnerabilities determines if the induced stress responses are successful and safe. If the stress management capacities are insufficient, the different stimuli may have antagonistic effects or inhibit beneficial adaptations. The selection of combinations for optimal adaptation is an important challenge for future research.

## Background

Exercise training is the systematic performance of physical activity for maintaining or improving domains of health-related physical fitness. The best studied and arguably most robust predictor of health-related physical fitness is aerobic capacity or cardiorespiratory fitness (CRF). CRF is measured as maximal oxygen uptake (VO_2_max), usually during a ramped exercise test performed to volitional fatigue (or exhaustion), and can be best reflected by the Fick principle whereby VO_2_max is the product of cardiac output (or Q representing blood flow or delivery) and the extraction of O_2_ between the arterial and venous side of activated tissue (_a-v_O_2_ difference) (Strasser and Burtscher, 2018). VO_2_max is considered the best indicator of endurance exercise capacity, strongly predicts morbidity and mortality, and is increasingly applied as a diagnostic tool in clinical practice. It has been consistently linked with better brain morphology in older adults and neurological disease, e.g., based on magnetic resonance imaging-assessed cortical thickness (Olivo et al., 2021) or cerebral myelination (Faulkner et al., 2024).

The brain is one of the most metabolically expensive organs, accounting for more than 20% of the resting energy expenditure of the body, despite constituting only 2% of the body mass (Padamsey and Rochefort, 2023). Neurons in the brain depend critically on O_2_ delivery for energy metabolism based on adenosine triphosphate (the primary cellular energy carrier molecule) production through oxidative phosphorylation, and there may be limited capacity for non-aerobic energy metabolic pathways (such as glycolysis) (Yellen, 2018). Therefore, the brain requires a continuous and highly regulated supply of O_2_. During intense exercise, there is a systematic process of blood flow regulation in the human body, and redistribution largely occurs based on the metabolic demands of activated tissues. Indeed, the O_2_ flux can increase up to 100-fold in skeletal (usually > 1/3 of the body mass) and cardiac (< 1% of the body mass) muscle, yet O_2_ consumption in the brain is maintained and does not increase substantially during exercise (Radak et al., 2013). This greatly reduces the relative O_2_ consumption of the brain. To that end, regulatory mechanisms, such as increasing cerebral blood flow, are necessary for providing an adequate supply of O_2_ and other nutrients within the brain during intense exercise.

Aerobic exercise training can effectively enhance cerebral functions, such as cognition, memory, and executive processing (Singh et al., 2025), perhaps by increasing O_2_ transport and uptake. Studies consistently demonstrate that regular aerobic exercise and higher CRF decrease the risk for various neurological disorders, including neurodegenerative diseases such as Alzheimer’s disease (Iso-Markku et al., 2022), Parkinson’s disease (Burtscher et al., 2025c), or multiple sclerosis (Motl et al., 2017), as well as in mental health disorders, including anxiety and depression (Kandola et al., 2019) and schizophrenia (Scheewe et al., 2013). Exercise may slow down neuropathological developments on several levels, for example, by lowering pathologically misfolded or aggregated protein levels (e.g., plasma tau levels (Raffin et al., 2025), slowing neuronal cell loss (e.g., as reported for Parkinson’s disease; Johansson et al., 2022), and cognitive decline (Tsukita et al., 2022). Functional imaging data even suggest that a mixed intense exercise program reversed neurodegenerative processes in Parkinson’s disease, preventing further decreases of dopamine neuron functions and neuromelanin levels (de Laat et al., 2024). Moreover, higher levels of exercise are robustly associated with reduced incidence and mortality rates in neurodegenerative diseases (Yoon et al., 2021; Iso-Markku et al., 2022; Livingston et al., 2024; Wang et al., 2025).

In contrast, impairments in O_2_ uptake, transport, and metabolism are common in neurological disorders such as Alzheimer’s disease (Liu et al., 2023), Parkinson’s disease (Burtscher et al., 2025a), and multiple sclerosis (Burtscher et al., 2025b), and in mental health disorders such as depression and anxiety. This could explain why improving CRF and oxygen transport through aerobic exercise training may reduce the risk of disease worsening. There is increasing evidence that specifically “training”, the cellular and organism capacity by reducing O_2_ availability (i.e., hypoxia) has great potential in Parkinson’s disease (Burtscher et al., 2025a), multiple sclerosis (Burtscher et al., 2025b), and other neurological (Burtscher et al., 2021) and mental health diseases. Such hypoxic training involves controlled exposure to continuous or intermittent mild hypoxia and is termed “hypoxia conditioning.” Hypoxia conditioning has been combined with aerobic exercise programs (Burtscher et al., 2025b), and may yield additional benefits, yet this remains controversial. We hypothesize that both interventions (aerobic exercise training and hypoxia conditioning) rely on partially overlapping molecular mechanisms (Butt et al., 2021; Ehrenreich et al., 2023), and this might support the premise of synergistic benefits through combinatory approaches of exercise training and hypoxia conditioning. Moreover, some physiological responses are relatively specific for one modality (aerobic exercise or ambient hypoxia) and may result in additive benefits when combined.

Taken together, we present the working hypothesis that transient tissue hypoxia resulting from increased oxygen demand, for example, during exercise (but further during elevated activities in other tissues, e.g., high cerebral activity), may be a crucial component contributing to the neuroprotective effects of exercise. In this mini-review, we synthesize current knowledge on the converging and diverging molecular pathways activated by exercise and hypoxia, with a focus on neuroprotective processes. By identifying relevant signaling axes, we highlight the potential that these interventions might be combined or be employed as complementary approaches for maintaining brain and mental health and for delaying neurodegenerative disease onset and progression.

## Search Strategy

This review is based on an extensive literature search primarily in Medline/PubMed and Google Scholar, using different combinations of the search terms hypoxia/hypoxic conditioning, therapeutic hypoxia, intermittent hypoxia, exercise, physical activity, cardiorespiratory fitness, neurological disorders, neurodegeneration, neuroprotection, neuropsychiatric diseases, cognition, and mental health. The relevant findings and literature, along with the large private repositories of the co-authors, were used to formulate and substantiate the working hypothesis: that increased oxygen demand during exercise may be a stimulus for neuroprotection. This hypothesis assumes that ambient hypoxia and aerobic exercise may share mechanisms revolving around hypoxia responses, which induce beneficial brain effects. The brain benefits of regular exercise and high CRF in humans are well-known (see background section), but similar effects elicited by hypoxia conditioning are only beginning to emerge. Therefore, the first step of this review was to assess the evidence that controlled hypoxia interventions positively affect brain functions.

To identify available data from clinical studies on hypoxia conditioning with outcomes on cerebral functions (for **Table 1** in the following section), the following key words were used in PubMed: (“therapeutic hypoxia” OR “hypoxia conditioning” OR “hypoxia hyperoxia” OR “hypoxia therapy” OR “intermittent hypoxia”) AND (neurolog* OR cogniti* OR dementia OR neurodegenerati*).

**Table 1:** Studies on intermittent hypoxia conditioning and outcomes related to human brain functions

Population and *n* (mean age ± SD)	Hypoxic dose	Functional neurological outcomes	Molecular, neurophysiological outcomes	Reference/note
RCT: 50 healthy participants in the hypoxia group (36 ± 7 yr), 50 healthy participants in the normoxia group (35 ± 6 yr).	2 sessions/day of 4 × 10 min cycles of F_I_O_2_ = 0.13, interspersed with 5 min normoxic cycles, for 5 d.	No differences in several cognitive tests: Digit Span Test; Digit Span Test reverse; Stroop Color-Word Test; Trail Making Test.	Improved cerebral blood flow after hypoxia: higher peak systolic blood flow velocity, cerebrovascular conduction, lower cerebrovascular resistance.	Zhang et al., 2025
Pilot study: 13 healthy and 7 subjects (27 ± 7 yr) with depression and/or autism spectrum disorder (6 major depression, 2 double diagnosis), all received motor-cognitive training in hypoxia (no control group).	Motor-cognitive training at F_I_O_2_ = 0.13 for 3.5 h daily for 3 wk.	Improved visual and verbal learning, processing speed, selective attention, problem solving and working memory. Improvements of depressive and anxiety symptoms in multiple tests (BSI, BDI, and HAMD).	Increased erythropoietin levels in serum; no consistent changes in erythrocyte and hematocrit. No adverse immunological alterations.	Mennen et al., 2024; Placebo and training effects will be controlled in future studies.
RCT: crossover study in 10 patients (between 41 and 75 yr; age only provided in 5-year age groups) with relapsing-remitting multiple sclerosis.	Acute intermittent hypoxia: 15 alternating exposures of 60 s to F_I_O_2_ = 0.09, interspersed with normoxic periods.	Acute intermittent hypoxia improved cognitive processing speed as assessed with the Symbol Digit Modalities Test, no changes in auditory/verbal memory.	Not assessed.	Sandhu et al., 2024; Preprint publication.
RCT: 14 (84 ± 5 yr) geriatric patients in hypoxia group, 11 (86 ± 6 yr) in sham group. All performed 6 wk of aerobic training 3×/wk for 20 min daily.	Intermittent hypoxic (F_I_O_2_ = 0.10–0.14, 1–5 min) and hyperoxic (F_I_O_2_ = 0.30–0.40, 1–3 min) periods for 30 min sessions prior to exercise. Protocols were customized to the subjects.	No significant interaction effect for Dementia Detection Test, medium effect size for improved performance of IHHC group in the clock drawing test.	Not assessed (but no changes in BDNF in a similar setup, in which no functional outcomes were tested (Behrendt et al., 2024)).	Behrendt et al., 2022b
Pilot study: 16 patients with MCI and 13 age-matched healthy elderly subjects (52 to 76 yr) were divided in the following groups: Healthy + Sham (n = 7), Healthy + IHHT (n = 6), MCI + Sham (n = 6), or MCI + IHHT (n = 10).	IHHT: 4 cycles of 5 min hypoxia (F_I_O_2_ = 0.12) interspersed with 3 min periods of hyperoxia (F_I_O_2_ = 0.30), 5 d/wk for 3 wk (15 sessions in total).	Slightly, but significantly improved MoCA scores in MCI + IHHT group only after the first day of IHHT.	Improved latency of cognitive evoked potentials in IHHT, increased APP110, GDF15 expression and MMP9 activity in all IHHT groups, reduced NETs formation and amyloid β expression in MCI + IHHT.	Serebrovska et al., 2022
Pilot study: 7 patients with amnestic MCI, all exposed to hypoxia (69 ± 3 yr).	IHC: 5 min F_I_O_2_ = 0.10, interspersed with 5 min normoxia; 8 cycles/session, 3 sessions/wk for 8 wk.	Improvements in mini-mental status exam and digit span scores.	Increased cerebral vasodilation and tissue oxygenation after hypoxia.	Wang et al., 2020
RCT: Multimorbid patients, 18 in the hypoxia group (81 ± 8 yr); 16 in the normoxia group (83 ± 6 yr). All received multimodal rehabilitation (2–3×/wk, 16–20 sessions in total).	IHC: 4–6 min F_I_O_2_ = 0.12, interspersed with 1–2 min F_I_O_2_ 35% (IHHC), the duration was adjusted to individual hypoxia sensitivity. 2–3 ×/wk, 15–16 session in 5–6 wk.	Improved cognitive performance only in IHHC group, as suggested by dementia test and clock drawing test.	Not assessed.	Bayer et al., 2017
RCT: Spinal cord injury patients, 17 in the hypoxia group (41 ± 17 yr); 16 in the normoxia group (42 ± 17 yr). All received body weight–supported treadmill training for 4 wk.	IHC: 15 cycles per day with F_I_O_2_ = 0.12 for 90 s, interspersed with normoxic periods (or continued normoxia in the control group) for 5 d, then 3 × per wk for 3 wk.	Improved verbal memory performance for immediate, short- and long-term recall in the IHC group compared to baseline, but not to control. No difference in the Rey–Osterrieth Complex Figure Test (visual memory, visuospatial constructional ability).	Not assessed.	Navarrete-Opazo et al., 2016
RCT: Physically active participants: 18 in the hypoxic group (66 ± 3 yr); 18 in the normoxia group (68 ± 4 yr). Both groups performed 30 min aerobic bicycle training 3× per wk	IHC: SpO_2_ 90%–85% in wk 1, 80% thereafter using NH, 90 min/session, 3 × per wk for 4 wk	Significantly improved cognitive function (Stroop test, word-color-task) in the hypoxic group	No increase of serum BDNF levels	Schega et al., 2016
Pilot study: Healthy, inactive participants; 17 in the hypoxic group (64 ± 3 yr), 17 in the normoxic group (64 ± 3 yr), Both groups performed regular 30 min full body strength-endurance training for 6 wk	10 min NH interspersed with 5 min normoxia: 4 cycles per day, aiming at reducing SpO_2_ stepwise: 90% in wk 1–2, 85% in wk 3 and 80% in wk 4–6)	Improved cognitive performance of the hypoxic group, indicated by significant differences in the d2 test (processing speed and attention), no significant difference in the Number Combination Test	Not assessed	Schega et al., 2013

BDI: Beck depression inventory; BDNF: brain-derived neurotrophic factor; BSI: brief symptom inventory; ERP: event related potential; F_I_O_2_: fractional inspired O_2_; HAMD: Hamilton Depression Rating Scale; IHC: intermittent hypoxia conditioning; IHHC: intermittent hypoxic hyperoxic conditioning; IHHT: intermittent hypoxia–hyperoxia training; MCI: mild cognitive impairment (mild cognitive disorder in DSM-V); MoCA: Montreal Cognitive Assessment test; NETs: neutrophil extracellular traps; NH: normobaric hypoxia; RCT: randomized controlled trial; SpO_2_: O_2_ saturation.

Inclusion criteria were: (i) approaches qualifying as hypoxia conditioning, (ii) inclusion of neurological (e.g., cognitive, neurophysiological, neuropathological) outcomes, and (iii) studies classified in PubMed as clinical trials (including pilot studies) or randomized controlled trials. Exclusion criteria were missing characterization of the hypoxic dose or breathing interventions, intermittent/continuous hypoxia only due to illnesses such as obstructive sleep apnea (as opposed to controlled artificial ambient hypoxia or altitude exposures), or experimental approaches in animals only.

The results were complemented with relevant studies identified through the general literature search of the review. Since experimental approaches of hypoxia conditioning (using controlled hypoxia in laboratory settings) in humans are more feasible for intermittent hypoxia conditioning approaches, only those were included in **Table 1**. Studies evaluating acute and prolonged continuous hypoxia exposures on human brain functions are discussed in the main text of the following sections.

## How Hypoxia Affects Brain Functions

The brain is highly dependent on a sufficient supply of oxygen, and even short periods of hypoxia can impair brain functions, depending mainly on the decreasing arterial partial pressure of O_2_ (McMorris et al., 2017). This is well known for cognitive functions, which rapidly deteriorate during exposures to sufficiently severe hypoxia. In natural environments, hypobaric hypoxia occurs at high altitudes, rendering such conditions ideal study environments for the effects of hypoxia, including on the brain. The hypoxia at high altitude is due to a smaller barometric pressure and the associated reduced partial pressure of O_2_, while the concentration of O_2_ in the air remains essentially the same as compared to sea level conditions. For clinical, research, and athletic training purposes, normobaric altitude chambers or devices for the inspiration of hypoxic gas mixtures are often applied. In such cases, the concentration of O_2_ is reduced, often described as the fraction of inspired oxygen (F_I_O_2_), which is approximately 0.21 at sea level. For better comparability, below are the corresponding F_I_O_2_ values for reported altitude levels of findings from high-altitude studies.

As recently reviewed (Aboouf et al., 2023), acute exposures to altitudes below 4000 m (4000 m corresponds to an F_I_O_2_ ≈ 0.135) rarely result in pronounced cognitive impairment, whereas higher altitudes can massively impair various cognitive domains. Among them, psychomotor function and long-term memory appear to be compromised more readily in hypoxic environments than information processing, working memory, and language (Jung et al., 2020; Su et al., 2024). Even very short exposures to altitudes of 5000 m (F_I_O_2_ ≈ 0.12) can significantly reduce reaction times, which is of considerable importance to consider, especially for pilots or mountain rescue personnel (Falla et al., 2024). Prolonged exposures to hypoxia/altitude increase the risk for deficits in physiology or function. For example, minor potential impairments have been observed after exposure of lowlanders already to a moderate altitude of 2260 m (F_I_O_2_ ≈ 0.17) for months to years, including slightly impaired visual construction (Zhang et al., 2011) and possibly altered brain connectivity (Chen et al., 2022). However, most of the many investigated functional parameters remained unchanged, and moderate altitudes (1000–2500 m, corresponding to F_I_O_2_ ≈ 0.18–0.16) are largely considered to promote health, including brain health (Burtscher et al., 2025c).

Exposures to acute or chronic hypoxia can impair some brain functions, but under certain circumstances, hypoxia can induce substantial benefits (Burtscher et al., 2021). This two-edged quality of hypoxia is likely related to its “hormesis”-like properties. Hormesis refers to a biphasic effect, meaning that a stimulus at a sufficiently low dose may enhance resilience, while a high dose can cause damage. Physiological responses to hypoxia may improve cellular metabolism and redox control, reduce inflammation, and enhance systemic oxygen transport by increasing vascularization and hemoglobin mass (Burtscher et al., 2022a).

Determinants of cerebral outcomes of hypoxia are mainly individual vulnerabilities and the hypoxic dose (Raberin et al., 2023). The hypoxic dose is defined by the severity of hypoxia and the duration of exposure. Notably, the frequency and patterns of exposure play an important role, too. Relevant differentiations are intermittent versus continuous hypoxia and acute *versus* chronic hypoxia. Residence at moderate or high altitude is an example of chronic continuous hypoxia, whereas a trek or hike at high altitude may be classified as acute continuous hypoxia exposure. Chronic continuous hypoxia has emerged as a promising condition for several neurological diseases, including multiple sclerosis, Parkinson’s disease, and Leigh syndrome, as demonstrated in rodent models, often using an F_I_O_2_ = 0.11, which corresponds to an altitude well above 5000 m (Burtscher et al., 2025b; Marutani et al., 2025; Rogers and Mootha, 2025). Importantly, such altitudes are probably associated with too severe hypoxia for health-promoting permanent residence of humans (Champigneulle et al., 2023; Burtscher et al., 2025b). In contrast, living at moderate altitudes has repeatedly been associated with improved health and reduced mortality from all causes (mainly from cardiovascular diseases) and may promote healthy aging (Burtscher and Samaja, 2024; Burtscher et al., 2025c).

In humans, the use of intermittent hypoxia for therapeutic purposes is gaining increasing interest and may be useful to treat cardiovascular, metabolic, musculoskeletal, and many other diseases, besides neurological disorders (Vose et al., 2022; Burtscher et al., 2024c). Controlled hypoxia interventions can be used for pre-acclimatization, thereby preventing adverse hypoxia consequences in a dose-dependent manner (Storz and Bautista, 2022; Mallet et al., 2023). Such methods reduce the risk of developing high altitude illnesses such as acute mountain sickness (e.g., Zhang et al., 2022) and the negative effects of hypoxia/altitude on cognition (e.g., Patrician et al., 2019). One study demonstrated that pre-acclimatization (7×1-hour F_I_O_2_ = 0.126, corresponding to 4500 m) reduced the number of risky decisions during a subsequent 12-hour exposure to F_I_O_2_ = 0.126 compared to a sham control group (Niedermeier et al., 2017). Additionally, detrimental effects of hypoxia have been described for learning (Pagani et al., 1998) or verbal memory (Nelson, 1982; Pelamatti et al., 2003; Wilson et al., 2009). Thus, exposure to severe hypoxia can acutely compromise several brain functions, notably reaction time, decision making, learning and memory, and other cognitive functions that are highly relevant to secure safety for professional (e.g., rescue operations) and leisure activities performed at altitude/in hypoxia. Moreover, exposures to severe hypoxia can be potential triggers of neurological and psychiatric symptoms, for example, inducing Parkinsonism in rare cases as suggested by several case studies (Swaminath et al., 2006; Park and Yang, 2013; Hur, 2015) or by triggering anxiety and depressive symptoms. Accordingly, severe hypoxia, by having pronounced effects on the cellular biochemistry, may result in pathological changes associated with neurological diseases, particularly with neurodegenerative diseases, such as Alzheimer’s dementia (Correia et al., 2013), Parkinson’s disease (Burtscher et al., 2021a; Janssen Daalen et al., 2024a). The direct effects of hypoxia on mitochondrial physiology, redox regulation, and inflammation modulate central shared molecular hallmarks of neurodegenerative disease (Burtscher et al., 2022; Wilson et al., 2023). However, hypoxia further influences another primary pathological event in neurodegeneration, namely protein aggregation pathology. There is strong evidence that brain hypoxia can promote amyloid-beta pathology in Alzheimer’s disease and alpha-synuclein misfolding and aggregation in Parkinson’s disease (Salminen et al., 2017). Based on the assumed biphasic properties of hypoxia (hormesis), it could be speculated that these adverse events occur, if the hypoxic stress is too severe for the innate protective mechanisms to be sufficiently activated in vulnerable brains. In contrast, mild hypoxia may “condition” the brain, conferring greater resilience and thereby protecting it from future hypoxic insults and probably other injurious challenges as well. This approach currently appears especially promising for Parkinson’s disease, with clinical trials investigating the potential of controlled intermittent hypoxia exposures for therapeutic purposes ongoing (Janssen Daalen et al., 2024b) and with recent results indicating the potential of chronic continuous hypoxia (F_I_O_2_ = 0.11, > 5000 m) to attenuate motor symptoms, alpha-synuclein pathology and neurodegeneration in animal models of Parkinson’s disease (Marutani et al., 2025).

Therapeutic intermittent hypoxia interventions are commonly referred to as intermittent hypoxia conditioning (IHC) and must be differentiated from chronic intermittent hypoxia. The term “chronic intermittent hypoxia” is commonly used to describe hypoxia patterns associated with detrimental health outcomes and can refer to hypoxic episodes in obstructive sleep apnea or experimental protocols mimicking such episodes. In a visionary review, Navarrete-Opazo and Mitchell (2014) categorized intermittent hypoxia protocols already as beneficial *versus* pathogenic based on the severity and frequency of the hypoxia. The analyses revealed that some protocols involving short hypoxia interventions with F_I_O_2_ > 0.09 (corresponding to an altitude of approximately 7000 m) and < 15 cycles per day often induce health-promoting effects, whereas more severe hypoxia and especially in combination with more than 48 cycles per day usually results in detrimental health effects (Navarrete-Opazo and Mitchell, 2014). The hypoxic cycles for therapeutic applications often are 4–8 minutes long and are interspersed with normoxic (F_I_O_2_ = 0.21, IHC) or hyperoxic (usually F_I_O_2_ = 0.3–0.4, intermittent hypoxia hyperoxia conditioning or IHHC) periods. Alternatively, even more severe hypoxia (down to F_I_O_2_ = 0.08) has recently been applied in approaches termed therapeutic acute intermittent hypoxia (tAIH), wherein shorter cycles (often 60–90 seconds) are interspersed with normoxic cycles of 30–120 seconds (for a total time of approximately 30 minutes). Such approaches have successfully improved respiratory and motor function in stroke patients (Hornby et al., 2024; Pearcey et al., 2025). tAIH has emerged as an effective strategy in neurological disorders/injuries compromising motor function, such as spinal cord injury (Tan et al., 2021), stroke (Hornby et al., 2024; Pearcey et al., 2025), or amyotrophic lateral sclerosis (Sajjadi et al., 2022), and the history and future of translational efforts and challenges have recently been summarized (Vose et al., 2022). Originally investigated for its potential to modulate respiratory motor plasticity in rodent models (Fuller et al., 2003; Golder and Mitchell, 2005), with the potential to restore breathing capacities in spinal cord injury, tAIH is now known to elicit plasticity in other neuronal circuits in animals and humans. This has been described notably for systems of motor control, with one session of tAIH (several cycles delivered on one day) sometimes being sufficient to yield immediate results (Vose et al., 2022). The neuroprotective properties of tAIH may be related to a brain-region-specific upregulation of neurotrophic factors, while not increasing neuroinflammation, as might be expected from pathological chronic intermittent hypoxia, at least in rats (Peters et al., 2015).

Despite a wealth of data from animal models (Navarrete-Opazo and Mitchell, 2014), evidence of therapeutic interventions to improve brain functions in humans remains scarce, and the risk of bias of most such studies is moderate to high, as suggested by bias assessments in recent systematic reviews (Behrendt et al., 2022a; Damgaard et al., 2023; Boulares et al., 2024).

The (largely adverse) effects of continuous hypoxia on cognitive functions have recently been reviewed (Ramírez-delaCruz et al., 2024). **Table 1** reports on outcomes on brain functions of mild intermittent hypoxia protocols that were designed as therapeutic interventions (hypoxia conditioning) for humans.

In contrast to the detrimental effects of severe acute hypoxia on the brain, but in accordance with the hormesis principle, several studies confirm the beneficial effects of controlled intermittent hypoxia on human brain functions. Repeated cycles of short exposures (usually 1–5 minutes) to hypoxia of F_I_O_2_ = 0.13–0.09, and interspersed with normoxic or hyperoxic periods, often resulted in clear cognitive benefits, when sessions took place regularly for 3–8 weeks (**Table 1**). This was true for people with and without pre-existing cognitive impairments. A recent pilot study observed improvements when motor-cognitive training was performed at much longer time in hypoxia (F_I_O_2_ = 0.13): for 3.5 hours per day for 3 weeks (Mennen et al., 2024). Although important controls (e.g., for placebo and training effects) have not yet been published, this study provides very promising results not only on the improvement of cognitive parameters, but also of psychiatric symptoms (depressive, anxiety, and obsessive-compulsive symptoms) (Mennen et al., 2024), suggesting intermittent hypoxia interventions as potential treatments for all kinds of neuropsychiatric diseases.

Among the observed benefits of different intermittent hypoxia conditioning protocols were improved verbal memory performance and learning (Navarrete-Opazo et al., 2016; Mennen et al., 2024), higher Montreal Cognitive Assessment test scores only acutely after the first hypoxia session (Serebrovska et al., 2022), higher digit span scores (Wang et al., 2020), improved performance in mini-mental status exam (Wang et al., 2020), clock drawing test (Bayer et al., 2017; Behrendt et al., 2022b), and better cognitive processing speed (Mennen et al., 2024; Sandhu et al., 2024).

Unsurprisingly, relatively few insights can be derived from the summarized studies on the mechanistic aspects of brain benefits due to intermittent hypoxia, as the investigation of molecular pathways and connectivity in the human brain is more difficult than in animal models. However, improved cerebral blood flow and oxygenation as well as favorable electrochemical adaptations may play a role (Wang et al., 2020; Serebrovska et al., 2022). Circulating brain-derived neurotrophic factor levels appear to not be modulated strongly in some intermittent hypoxia interventions (Schega et al., 2016; Behrendt et al., 2024), potentially speaking against an important role of neurotrophic factor upregulation as a result of hypoxia exposure, although this remains to be investigated in more detail: certainly not only the type of intermittent hypoxia, but also the type of neurotrophic factor and the methodological assessment and anatomical localization are important considerations for future studies. Higher hypoxic doses, such as in the protocol explored by Mennen and colleagues (F_I_O_2_ = 0.13 for 3.5 hours per day for 3 weeks), likely involve an upregulation of erythropoietin (Mennen et al., 2024). This effect would traditionally not be expected to contribute to brain benefits in hypoxia conditioning protocols with short cycles, since the cumulative hypoxia exposure is not thought to be sufficient to induce erythropoietin upregulation, when comparing to the hypoxic dose needed for continuous hypoxia to modulate erythropoietin levels (Treff et al., 2022). However, recent results indicate that short cycles of hypoxia with a sufficiently low F_I_O_2_ stimulate erythropoiesis. Specifically, reducing the arterial oxygen saturation of young adults to 79% (an F_I_O_2_ = 0.103 ± 0.007 was necessary to achieve this) during 8 cycles of 5 minutes (interspersed with 2 minutes of normoxia, thus only 40 minutes of hypoxia in total) elicited a similar erythropoietin response, such as the exposure to 120 minutes continuous hypoxia (Wojan et al., 2021). Increasing erythropoietin levels are an important aspect of the assumed benefits of high-altitude training (Płoszczyca et al., 2018; Bonato et al., 2023) and has great neuroprotective potential in the brain (Wakhloo et al., 2020; Butt et al., 2021; Ehrenreich et al., 2023). Therefore, modulation of erythropoietin may be a mechanism underlying many intermittent hypoxia conditioning approaches.

No improvements in cognitive effects were observed in a study using 2 sessions of 4 × 10 minutes cycles of F_I_O_2_ = 0.13 per day (5 minutes normoxic cycles in between), for 5 days, despite increased cerebral blood flow after the hypoxia exposure (Zhang et al., 2025). There may be several reasons why cognitive abilities did not benefit in this study. First, unlike most of the other studies presented in **Table 1**, the subjects were healthy, young adults. Moreover, the hypoxia intervention only lasted for 5 days, which may be too short to elicit meaningful adaptations, especially as an F_I_O_2_ = 0.13 represents relatively mild hypoxia in comparison to the hypoxia used in other studies.

Overall, intermittent hypoxia can improve brain functions, but probably this approach is more effective in people with declining cognitive functions. One crucial factor determining the efficacy and safety is the hypoxic dose. Most studies in **Table 1** repeatedly exposed the subjects for 1–5 minutes to hypoxia of F_I_O_2_ = 0.13–0.09, which was tolerated very well in all studies. Even performing motor-cognitive training at an F_I_O_2_ = 0.13 for 3.5 hours was not associated with any adverse events (Mennen et al., 2024). These results suggest that the threshold for intermittent hypoxic cycles (of maximum several minutes) of approximately F_I_O_2_ = 0.09 as described by Navarrete-Opazo and Mitchell (2014) may be accurate for beneficial effects on the brain, with milder hypoxia allowing more cycles. Greater hypoxic doses may result in detrimental effects. It is important to note that the duration plays a crucial role: if the exposure to hypoxia cycles exceeds several minutes, already at an altitude of 5000 m (F_I_O_2_ ≈ 0.12), there can be negative cognitive effects. The importance of the normoxic and/or hyperoxic regeneration cycles should not be neglected. Although those are considered to be important for some of the beneficial adaptations of intermittent hypoxia, for example, due to the upregulation of oxidative stress and anti-inflammatory responses (Burtscher et al., 2022), both are probably crucial for people exposed to hypoxia to better tolerate the hypoxic stress.

Based on the described neuroprotective potential of inspiratory hypoxia, the question arises whether tissue hypoxia as a result of increased oxygen demand due to intense aerobic exercise may contribute to the brain benefits conferred by regular exercise.

## Hypoxia Sensing and Responses in Exercise

Hypoxia sensing mechanisms exist at cellular and systemic levels. At the cellular level, these notably include the well-characterized hypoxia-inducible factor (HIF) pathways. At normal oxygen levels (and in the presence of alpha-ketoglutarate, ascorbate, and ferrous iron), the oxygen-dependent enzymes HIF-prolyl hydroxylase domain proteins hydroxylate HIF alpha-subunits, marking them for degradation by the proteasome (Kaelin and Ratcliffe, 2008). In hypoxia, the HIF alpha-units stabilize, dimerize with HIF beta-subunits, and coordinate a large transcriptional response that includes energy-metabolic adaptations (e.g., inhibition of oxidative phosphorylation, upregulation of glucose transport and glycolysis), changes in the blood and vascular system (e.g., upregulation of erythropoietin, angiogenesis), lipid metabolism adaptations, and the promotion of antioxidant and anti-inflammatory mechanisms (Burtscher et al., 2022). At a systemic level, peripheral chemoreceptors (mainly those in the carotid bodies) sense a low partial pressure of oxygen in the blood and transduce this information to brain stem circuits involved in regulating respiratory (i.e., the hypoxic ventilatory response) and cardiovascular responses (Iturriaga et al., 2021). Overall, systemic hypoxia leads to sympathetic activation and vasodilation across most tissues (except in the pulmonary vessels).

Interestingly, many effects similar to these acute hypoxia responses are elicited by exercise. This is likely due, in part, to local hypoxic conditions consequently to increased oxygen demand in primarily affected tissues, such as skeletal muscle and heart. Carotid bodies are sensitive to the partial pressure of oxygen in the blood as well as carbon dioxide and lactate (Iturriaga et al., 2021), all factors that change with intense exercise. Many physiological outcomes, including sympathetic activation, increased heart rate and cardiac output, increased ventilation and vasodilation, change similarly during aerobic exercise compared with environmental hypoxia. Like aerobic exercise, hypoxia is an important modulator of cerebral blood flow (Claassen et al., 2021), which may contribute to the neuroprotective effects of both interventions.

At the cellular level, hypoxia responses can be induced by muscle contraction and the resulting increased oxygen demand (Lindholm and Rundqvist, 2016). Accordingly, HIF pathways can be activated in exercising muscles, but more surprisingly, exercise can result in HIF upregulation in the brain. However, experimental evidence for this phenomenon is currently restricted to animal models. For example, in mice performing aerobic exercise, elevated HIF mRNA levels and the modulation of HIF-downstream target genes have been reported in the striatum and in the substantia nigra (Smeyne et al., 2015; Halliday et al., 2019). In mice, HIF1alpha has even been shown to be necessary for the neuroprotective effects of exercise (Smeyne et al., 2015).

HIF-mediated effects may contribute to the positive effects of exercise on synaptic function and neuroprotection to some extent, yet many questions regarding the role of hypoxia response systems in neuroprotection remain unanswered. For instance, it remains unclear how hypoxia signals are transmitted from the primarily affected tissues (e.g., skeletal muscle and heart) to the brain; whether HIF activation in response to exercise occurs in the human brain; and whether it is primarily regulated at the transcriptional level or post-translationally via prolyl hydroxylase domains. Additionally, our knowledge on the optimal selection of timing, duration, intensity, and modality of exercise for optimizing central HIF activation is very limited. Such knowledge would be particularly important to avoid maladaptive inflammation or oxidative damage, which can be associated with HIF-activation and may be especially detrimental in people with neuropsychiatric and neurodegenerative disorders. Addressing these gaps in humans will require integrated approaches, combining muscle physiology, neuroscientific approaches such as cerebrovascular imaging, and the analysis of large sets of molecular biomarkers (e.g., OMICs approaches) in both healthy participants and clinical populations.

## Overlapping Molecular Hypoxia and Exercise Responses Potentially Affecting Neuroprotection

Controlled exposure to hypoxia and aerobic exercise can induce overlapping (but also distinct) acute responses and medium-term adaptations. The increased oxygen demand in contracting muscles during exercise acutely leads to an accumulation of HIFs and, in the long term, to a potentially more tightly regulated (and perhaps more sensitive) HIF response (Lindholm and Rundqvist, 2016). The health-promoting outcomes of the hypoxia response may ultimately be an increased resilience to hypoxic injury or the re-establishment of sufficient tissue oxygenation, for example, through mechanisms that increase the perfusion of hypoxic tissues. Supporting this idea, a recent rodent study indicates that aerobic exercise can eliminate transient and local occurrences of brain hypoxia, termed “hypoxic pockets” (Beinlich et al., 2024). The authors of that study applied novel techniques to monitor cortical partial oxygen tension in living and behaving mice and observed the spontaneous and transient formation of hypoxic pockets. It could be speculated that increasing accumulations of hypoxic pockets in the brain are involved in the development of brain pathologies and that resolving them is one of the beneficial effects of exercise (and hypoxia conditioning, by improving the management of hypoxic stress) on the brain.

**Figure 1:**
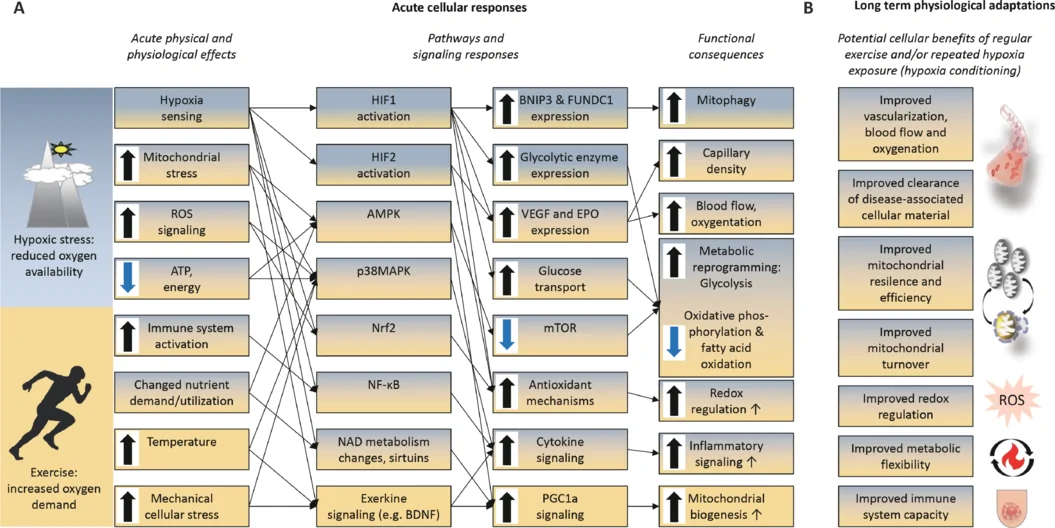
Overlaps in acute molecular responses (A) and adaptive processes (B) between hypoxia exposure and exercise with potentially beneficial outcomes on oxygen-linked processes in the brain. Yellow shading indicates processes mainly activated by exercise; gradients from gray to yellow indicate overlapping processes following both hypoxic stress and exercise bouts. AMPK: AMP-activated protein kinase; ATP: adenosine triphosphate; BDNF: brain-derived neurotrophic factor; BNIP-3: BCL2 interacting protein 3; EPO: erythropoietin; FUNDC1: FUN14 domain containing 1; HIF: hypoxia inducible factor; mTOR: mammalian target of rapamycin; NF-κB: nuclear factor kappa B; Nrf2: nuclear factor (erythroid derived 2)-like 2; NAD: nicotinamide dinucleotide; p38MAPK: p38 mitogen-activated protein kinases; PGC-1α: peroxisome proliferator-activated receptor gamma coactivator 1-alpha; ROS: reactive oxygen species; VEGF: vascular endothelial growth factor.

**Figure 1** depicts the overlap of acute molecular responses and physiological adaptations to exercise and hypoxia.

Energetic and mitochondrial stress can be consequences of hypoxia due to reduced ambient oxygen availability (such as at high altitude) or due to elevated oxygen demand. An increased oxygen demand may be a consequence of intense aerobic exercise and cause tissue hypoxia, especially in skeletal muscle and heart. The resulting decrease in oxygen availability is sensed by molecular and systemic mechanisms, leading to changes in blood flow (Claassen et al., 2021), including to and within the brain. Additionally, both exercise and hypoxia modulate the efficiency of the glymphatic system, the waste clearance system of the brain (Wang et al., 2023; Yoo et al., 2025). While severe hypoxia (such as occurs in obstructive sleep apnea) has been demonstrated to impair the glymphatic system, exercise training appears to improve it (Wang et al., 2023; Yoo et al., 2025). We hypothesize that hypoxia conditioning may also improve glymphatic function, but this remains to be experimentally demonstrated.

Mitochondrial stress resulting from exercise in affected tissues likely is involved in triggering the production and release of exercise-induced signaling molecules (“exerkines”) into the circulation, thereby affecting the physiology of even distant organs (Chow et al., 2022). It also increases the production of reactive oxygen species, which serve as signaling molecules, but can lead to harmful oxidative stress and activate inflammatory pathways, both of which are molecular hallmarks of neurodegenerative diseases (Sies and Jones, 2020). To counteract these potentially cell-damaging effects, various protective pathways are activated in response to both ambient hypoxia and exercise. These pathways notably include the activation of a series of transcription factors that coordinate cellular responses to hypoxia (HIFs), oxidative stress (nuclear factor (erythroid derived 2)-like 2), and inflammation (nuclear factor kappa B) (Kaelin and Ratcliffe, 2008; Done et al., 2016; Lindholm and Rundqvist, 2016; Burtscher et al., 2022). Energetic stress is a common consequence of inspiratory hypoxia and of intense or prolonged exercise, and activates the cellular energy sensor AMP-activated protein kinase, which downregulates anabolic pathways (e.g., inhibiting mammalian target of rapamycin) and contributes to the regulation of mitochondrial biogenesis (increasing mitochondrial mass) (Herzig and Shaw, 2018). While exercise is known to promote mitochondrial biogenesis (Memme et al., 2021), acute severe hypoxia can induce mitophagy (Burtscher et al., 2022), i.e., the autophagic degradation of mitochondria. HIF-1 can induce upregulation of mitophagy pathways involving the mitophagy regulating proteins BCL2 interacting protein 3 (BNIP3) and BCL2/adenovirus E1B 19 kDa protein-interacting protein 3-like (BNIP3L/NIX) (Sowter et al., 2001; Zhang et al., 2008). The mitochondrial outer membrane protein FUN14 domain containing 1 has been demonstrated to mediate mitophagy in hypoxia, via light chain 3 and autophagosome formation (Liu et al., 2012). Both effects, mitochondrial biogenesis and mitochondrial clearance, can be beneficial for cells: Mitochondrial biogenesis increases the density of functional mitochondrial networks, while mitophagy may clear dysfunctional mitochondria that can release reactive oxygen species, pro-inflammatory factors, and promote cell death. Importantly, mitophagy does not necessarily selectively clear dysfunctional mitochondria, and many different conditions can promote the clearance of even healthy mitochondria (Uoselis et al., 2023). Extreme altitude exposure of lowlanders (e.g., during an expedition on the Mount Everest, 8848 m), for example, may cause reduced mitochondrial mass of specific populations such as subsarcolemmal mitochondria (Murray, 2016), possibly independent of dysfunction. However, such effects were less pronounced in simulated altitudes, suggesting that other factors, such as physical exertion of extreme altitude expeditions, more importantly, may modulate mitochondrial mass more than hypoxia *per se.*

Other outcomes that are shared by repeated or prolonged exposure to hypoxia and regular aerobic exercise include the promotion of vascularization and capillarization (Burtscher et al., 2022; Mølmen et al., 2024), as well as an increase in hemoglobin and red blood cells. These effects are partly mediated by the HIF-regulated vascular endothelial growth factor and erythropoietin, which are both considered to have neuroprotective properties (Ehrenreich et al., 2023; Tari et al., 2025). In the short term, hypoxia and intense aerobic exercise cause a shift from aerobic to anaerobic metabolic pathways, enabling adenosine triphosphate production in conditions where oxygen is limited. While this effect may be limited in neurons (Yellen, 2018), it is an important mechanism in other brain cells, such as glial cells. It is possible that the repeated switching between metabolic pathways improves substrate utilization and overall metabolic flexibility in the brain, an effect that may be evoked by both exercise and hypoxia (Palmer and Clegg, 2022). However, conclusive experimental evidence for these effects is still lacking. Overall, environmental hypoxia and aerobic exercise acutely trigger overlapping mechanisms, promoting capillarization, oxygen transport in blood, as well as antioxidant and anti-inflammatory mechanisms. The acute effects on mitochondrial density may be different following ambient hypoxia or exercise, with hypoxia favoring mitophagy and exercise favoring the induction of mitochondrial biogenesis. It should be emphasized that the overview in **Figure 1** of the acute molecular effects of hypoxia and aerobic exercise is highly simplified. These effects strongly depend on the type and dose of hypoxia/exercise but also on individual differences and conditions, such as health, dietary status, and temperature.

Shared beneficial physiological adaptations to repeated or prolonged controlled mild hypoxia and regular aerobic exercise in the brain may include improved O_2_ transport (cardiovascular system) and utilization (mitochondria), mitochondrial health and redox regulation, metabolic flexibility, immune functions and inflammation regulation, waste product clearance, and enhanced cellular resilience. The combination of overlapping and specific effects of ambient hypoxia and exercise makes a combination of those interventions particularly interesting for harnessing synergies and additive effects. Research showing the important role of ‘functional hypoxia’ in mediating positive brain adaptations, such as neuroplasticity, neurogenesis, and neuroprotection, following motor and/or cognitive activity in hypoxic conditions supports this potential (Wakhloo et al., 2020; Butt et al., 2021; Mennen et al., 2024). Relatively specific physiological responses, such as exercise-related promotion of mitochondrial biogenesis and hypoxia-induced mitochondrial quality control mechanisms, such as mitophagy, may be especially interesting to take advantage of additive effects to control mitochondrial homeostasis.

Importantly, the adaptations to both hypoxia and exercise acutely represent cellular stressors. Therefore, their combination can also result in adverse effects, especially if either the hypoxic dose or the exercise dose is too high, or if insufficient regeneration time (or not enough/sufficient normoxic/hyperoxic periods between hypoxic intervals in intermittent hypoxia conditioning) is provided. Finally, the stress responses of one stimulus or both stimuli combined may impair the induction of beneficial adaptations through antagonistic or inhibitory effects. These possible outcomes must be considered in future research.

## Conclusions and Perspectives

In summary, both aerobic exercise training and hypoxia conditioning appear to be effective strategies for maintaining a healthy cerebral environment by increasing cellular and tissue resilience to insults from energetic crises or oxidative stress. Hypoxic episodes resulting from exercise may even account for some of the neuroprotective effects of aerobic exercise training, and mechanistic studies are needed to investigate this question. Regulation of cerebral blood flow may be a crucial mechanism for keeping the brain well perfused and clearing potentially harmful “hypoxic pockets” from the brain. Moreover, the possibly causal involvements of hypoxia and HIF activities in positive outcomes of exercise in the brain (including erythropoietin responses and vascular remodeling) suggest that inspiratory hypoxia could be an effective additional or alternative treatment strategy for many neurological diseases. As an alternative strategy, (passive) hypoxia exposure might be particularly useful to induce exercise-like adaptations in people unable or unwilling to perform the mechanical work necessary for exercise. As an adjunct to exercise therapy, inspiratory hypoxia may amplify hypoxic signals that are thought to be necessary for tissue adaptations in organs such as skeletal muscle, the vasculature, or the brain. The way exercise signals are transmitted to the brain from primarily affected tissues (such as skeletal muscle) remains unclear, but is likely to involve exerkines and systemic chemophysical changes. Although not yet fully understood, hypoxia sensing may be a central component of these signaling modalities. Cellular responses to hypoxic stress may be involved in exerkine production and release, for example, in muscle cells with acutely increased oxygen demand and energetic stress. Additionally, the regulation of systemic responses to changes in blood gases and pH by peripheral chemoreceptors and the cerebral blood flow response links hypoxic and exercise responses, inducing direct effects on the brain.

The combination of exercise and hypoxia is a promising modality to improve beneficial outcomes on brain in a synergistic or additive manner, it must be considered that both acute exercise and hypoxia exposure represent physiological stressors, challenging the cellular metabolic and biochemical homeostasis and systemic physiology. The respiratory, cardiovascular and immune systems are regulated strongly during both exercise and hypoxia. Regarding the hormesis principle, specific therapeutic windows in terms of exercise dose and hypoxic dose likely exist and inadequate doses may reduce the safety of related interventions, especially if combined. As such, the combination of exercise and hypoxic stimuli may counteract beneficial adaptations and their combination could even increase the risk of stress-related cellular damage and adverse systemic outcomes, including overtraining. The determination of optimal combination protocols, customized to individual resilience and vulnerabilities, remains challenges for future research.

## Data Availability

*Not applicable.*
